# The Genetic Basis of Gene Expression Divergence in Antennae of Two Closely Related Moth Species, *Helicoverpa armigera* and *Helicoverpa assulta*

**DOI:** 10.3390/ijms231710050

**Published:** 2022-09-02

**Authors:** Ping-Ping Guo, Guo-Cheng Li, Jun-Feng Dong, Xin-Lin Gong, Lingyu Wang, Ke Yang, Jun Yang, Ling-Qiao Huang, Chen-Zhu Wang

**Affiliations:** 1 State Key Laboratory of Integrated Management of Pest Insects and Rodents, Institute of Zoology, Chinese Academy of Sciences, Beijing 100101, China; 2 CAS Center for Excellence in Biotic Interactions, University of Chinese Academy of Sciences, Beijing 100101, China; 3 Forestry College, Henan University of Science and Technology, Luoyang 471000, China; 4 Department of Biology, Duke University, Durham, NC 27708, USA

**Keywords:** allele-specific expression, *cis*- and *trans*-regulatory variants, antennae, pheromone receptors

## Abstract

The closely related species *Helicoverpa armigera* (*H. armigera*) and *Helicoverpa assulta* (*H. assulta*) have different host plant ranges and share two principal components of sex pheromones but with reversed ratios. The antennae are the main olfactory organ of insects and play a crucial role in host plant selection and mate seeking. However, the genetic basis for gene expression divergence in the antennae of the two species is unclear. We performed an allele-specific expression (ASE) analysis in the antennal transcriptomes of the two species and their F_1_ hybrids, examining the connection between gene expression divergence and phenotypic differences. The results show that the proportion of genes classified as all *cis* was higher than that of all *trans* in males and reversed in females. The contribution of regulatory patterns to gene expression divergence in males was less than that in females, which explained the functional differentiation of male and female antennae. Among the five groups of F_1_ hybrids, the fertile males from the cross of *H. armigera* female and *H. assulta* male had the lowest proportion of misexpressed genes, and the inferred regulatory patterns were more accurate. By using this group of F_1_ hybrids, we discovered that *cis*-related regulations play a crucial role in gene expression divergence of sex pheromone perception-related proteins. These results are helpful for understanding how specific changes in the gene expression of olfactory-related genes can contribute to rapid evolutionary changes in important olfactory traits in closely related moths.

## 1. Introduction

Phenotypic differences between related species are of importance in speciation, reproductive isolation and adaptive evolution [1,2,3,4]. Therefore, the genetic basis of phenotypic differences between related species is a core issue of evolutionary biology [5]. Gene expression regulation is a crucial step in the transformation of genotypes into phenotypes, and regulatory variation is common among related species and contributes to phenotypic diversity [6]. Clarifying the genetic changes underlying these expression differences is important to understand the evolution of gene expression regulation and its role in phenotypic differentiation [7]. The improvement of methods for measuring gene expression enables the easier identification of gene expression divergence. Through interspecific hybridization and RNA sequencing (RNA-seq), allele-specific expression (ASE) in F_1_ hybrids can be used to determine the genetic basis of gene expression divergence in related species [8,9]. ASE refers to the characteristic of the preferential expression of a parental allele in a F_1_ hybrid owing to the variation in regulatory sequences of the parental genomes [10], which in association with the difference in gene expression between the parents, allows for the assessment of *cis*- and *trans*-regulatory variants. Studies show that *cis*-regulatory variants have a targeted effect and play an important role in phenotypic differences, whereas *trans*-regulatory variants have a pleiotropic effect and evolve under stronger selection constraints [6,11,12,13]. *cis*- and *trans*-regulatory variants play a crucial role in the differentiation of sexual dimorphism [14].

*H. armigera* (Hübner) and *H. assulta* (Guenée) are closely related species [15]. They have distinct differences in two phenotypes: (1) in both species, (*Z*)-11-hexadecenal (Z11-16:Ald) and (*Z*)-9-hexadecenal (Z9-16:Ald) are the principal sex pheromone components, but with opposite ratios: 98:2 and 5:95, respectively [16]. (2) Their host plant ranges are different: *H. armigera* is a typical polyphagous species, whereas *H. assulta* is an oligophagous species [17]. *H. armigera* and *H. assulta* can hybridize in the laboratory [18]. When *H. armigera* is the female parent, the initial crosses produce fertile males and two types of sterile abnormal individuals; when *H. assulta* is the female parent, the reciprocal crosses produce fertile males and females [18,19].

Antennae are the main olfactory organ of insects with typical sexual dimorphism [20,21]. The antennae of male moths are mainly used to sense the sex pheromones released by females for courtship and mating [22], the antennae of female moths mainly sense plant volatiles for host plant selection [23]. Therefore, studying the genetic basis of phenotypic differences in the antennae of closely related moths is of considerable importance for understanding the mechanism of prezygotic isolation and adaptive evolution. Some genes were dynamically expressed in antennae of insects. For example, *bric à brac*, a gene controlling sex pheromone choice in males of the Z-strain of *Ostrinia nubilalis*, was upregulated during early neuronal development in pupal antennae and reached a high expression level in adult antennae [24]. There are similarities and distinct differences in the physiological characteristics and gene expression of the antennae of *H. armigera* and *H. assulta* [25,26]. The olfactory sensory neurons (OSNs) responding to Z11-16:Ald and Z9-16:Ald are distributed in the male antennae of *H. armigera* and *H. assulta*, but in opposite ratios [27]. Correspondingly, HarmOR13 tuned to Z11-16:Ald and HassOR14b tuned to Z9-16:Ald are the most highly expressed pheromone receptors (PRs) in the male antennae of *H. armigera* and *H. assulta*, respectively [28,29]. Previous studies have shown that the functions of orthologous odorant receptors (ORs) in *H. armigera* and *H. assulta* are similar [30,31,32,33]. Thus, the difference in the antennal phenotype of the two species may be associated with gene expression divergence. However, the regulatory mechanisms of expression divergence of *PRs* and other genes in the antennae of *H. armigera* and *H. assulta* remain unclear.

To explore the genetic basis of gene expression divergence in the antennae, in this study, we used transcriptome sequencing to analyze the total transcriptional abundance and ASE of antennal genes of *H. armigera*, *H. assulta* and their F_1_ hybrids. Then, we investigated the inheritance modes of F_1_ hybrids and the contributions of *cis*- and *trans*-regulatory variants to the gene expression divergence of the parental antennae. The results reveal that the regulatory patterns in male antennae are different from those in the female antennae, abnormal expression of alleles in the sterile F_1_ hybrids would affect the inference of regulatory patterns, and *cis*-related regulations were associated with the evolution of phenotypic differences in the perception of sex pheromones in *H. armigera* and *H. assulta*.

## 2. Results

### 2.1. Interspecific Hybridization and Electrophysiological Responses of H. armigera and H. assulta

**Figure 1:**
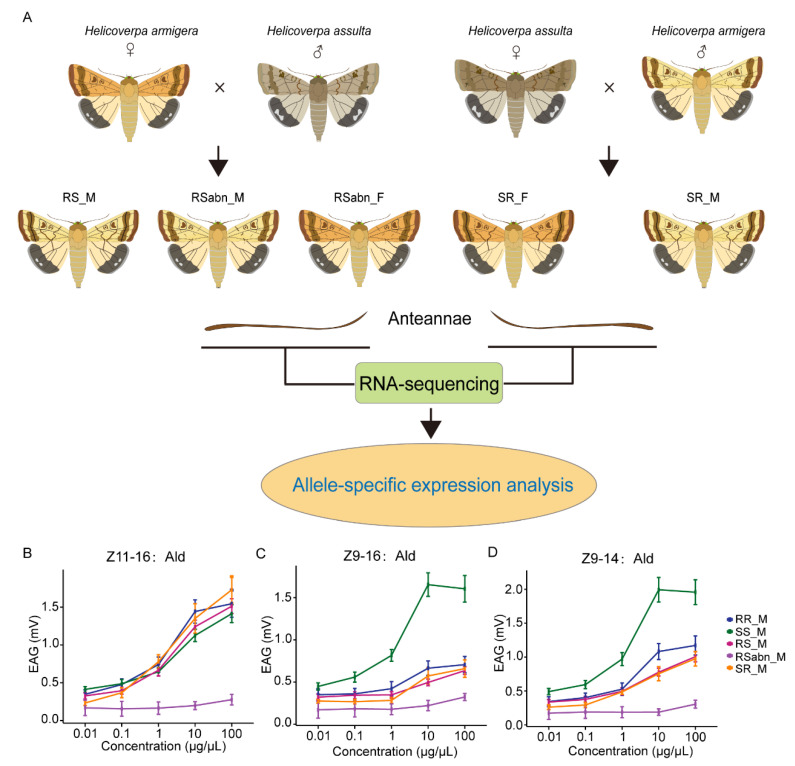
Interspecific hybridization and electrophysiological responses of *H. armigera, H. assulta* and F_1_ hybrids. (**A**) The initial crosses employed female *H. armigera* (RR_F) as the female parent and male *H. assulta* (SS_M) as the male parent, which produced three groups of F_1_ hybrids, comprising fertile males (RS_M), sterile abnormal males (RSabn_M) and sterile abnormal females (RSabn_F). The reciprocal crosses employed female *H. assulta* (SS_F) as the female parent and male *H. armigera* (RR_M) as the male parent, which produced two groups of F_1_ hybrids, comprising fertile males (SR_M) and fertile females (SR_F). The antennae of the parents and their F_1_ hybrids adults were used for transcriptome sequencing, and then ASE analysis was performed. (**B**–**D**) The dose-EAG responses of RR_M, SS_M, RS_M, RSabn_M and SR_M to Z11-16:Ald (**B**), Z9-16:Ald (**C**), and Z9-14:Ald (**D**).

When *H. armigera* is the female parent, the initial crosses produce fertile males (RS_M) and abnormal F_1_ hybrids (Figure 1A) [19]. The abnormal F_1_ hybrids were further divided into two groups based on the abnormal morphology of the pupae: RSabn_A and RSabn_B [19]. To identify the sex of the abnormal F_1_ hybrids, we examined the expression levels of two genes in these individuals: the Z chromosome marker gene *TPI* (triosephosphate isomerase) [34] and the W chromosome marker gene *GUW1* [35]. The expression levels of *TPI* and *GUW1* in RSabn_A were similar to those in RR_M and SS_M, and the expression levels of *TPI* and *GUW1* in RSabn_B were similar to those in RR_F and SS_F (Figure S1), indicating that RSabn_A is male and RSabn_B is female. When *H. assulta* is the female parent, the reciprocal crosses produce fertile males (SR_M) and fertile females (SR_F). Therefore, from the intraspecific and interspecific crosses of the two species, we obtained nine groups of insects, including five male groups (RR_M, SS_M, RS_M, RSabn_M, and SR_M) and four female groups (RR_F, SS_F, RSabn_F and SR_F) (Figure 1A). We collected the antennae of adult moths in each group, and prepared libraries for RNA-seq. Three replicates were set except for the SR_F group, where only two replicates were obtained because the number of SR_F were relatively limited.

We further analyzed the electrophysiological responses of the antennae of RR_M, SS_M, RS_M, RSabn_M, and SR_M to two sex pheromone components, Z11-16:Ald and Z9-16:Ald, and the behavioral antagonist (*Z*)-9-tetradecenal (Z9-14:Ald) by electroantennogram (EAG). The dose response curves of male antennae show that the antennae of RR_M, SS_M, RS_M, and SR_M all strongly responded to Z11-16:Ald (Figure 1B), and the responses of SS_M antennae to Z9-16:Ald and Z9-14:Ald were much stronger than those of the antennae of RR_M, RS_M and SR_M (Figure 1C,D). RSabn_M antennae had almost no response to these three compounds (Figure 1B–D).

### 2.2. Transcriptome Analysis of H. armigera, H. assulta and F_1_ Hybrids

We mapped the reads of the parents and F_1_ hybrids to the reference (see Materials and Methods) and identified genetic differences in coding sequences among the two species and F_1_ hybrids of the same sex. We identified > 2.2 million species-informative sites (53.44% of all single-nucleotide polymorphisms (SNPs)) in males (Table S1), and >1.9 million species-informative sites (49.90% of all SNPs) in females (Table S2). On this basis, we assigned 53.77% of reads in the transcriptomes of male F_1_ hybrids (Table S1) and 54.04% of reads in the transcriptomes of female F_1_ hybrids (Table S2) as originating from one or another parental genome using HyLiTE [36]. Next, the aligned reads were used to measure gene expression levels.

ASE analysis was performed to explore the expression pattern bias of parental genes in F_1_ hybrids. Although we used the *H. armigera* transcriptome as reference, the ASE analysis of five groups of F_1_ hybrids showed that expression was slightly biased in favor of *H. assulta* (Figure S2), indicating that the number of genes derived from *H. assulta* is slightly higher than that from *H. armigera* in all F_1_ hybrids, a possible reason for this is that more *cis*-regulatory changes are fixed in *H. assulta*.

### 2.3. Principal Component Analysis (PCA) of H. armigera, H. assulta and F_1_ Hybrids

**Figure 2:**
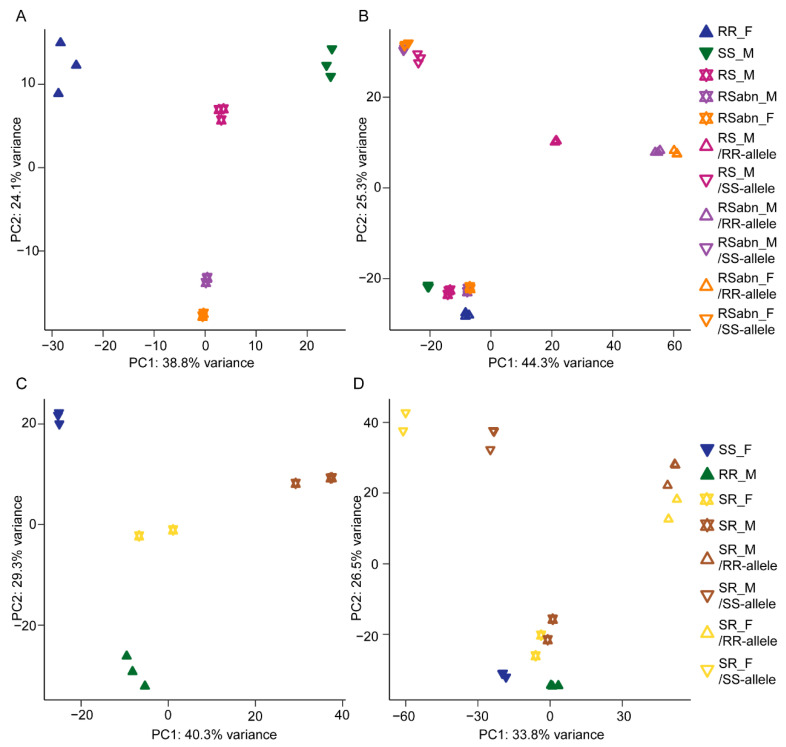
Principal component analysis (PCA) of the antennal transcriptome of *H. armigera*, *H. assulta* and their F_1_ hybrids. (**A**) PCA of the antennal transcriptome of RR_F, SS_M and their F_1_ hybrids. (**B**) PCA of the antennal transcriptome of RR_F, SS_M, their F_1_ hybrids and alleles within F_1_ hybrids. (**C**) PCA of the antennal transcriptome of SS_F, RR_M and their F_1_ hybrids. (**D**) PCA of the antennal transcriptome of SS_F, RR_M, their F_1_ hybrids and alleles within F_1_ hybrids.

To explore the variations in antennal gene expression among the two parents and F_1_ hybrids, we performed a PCA. In initial crosses, the variance between sterile F_1_ hybrids (RSabn_M and RSabn_F) and fertile individuals (RR_F, SS_M and RS_M) was large (Figure 2A). In reciprocal crosses, the variance between SR_M and SS_F was more than the variation between the two parents (Figure 2C).

We also explored the expression variance of alleles in F_1_ hybrids. In initial crosses, the results show that, compared with the alleles from *H. assulta*, the distribution of *H. armigera*-derived alleles expressed in sterile F_1_ hybrids and RS_M was scattered (Figure 2B). We annotated the top 20 genes that contributed to the variance of *H. armigera*-derived alleles expressed in RSabn_M and RSabn_F, and observed that these genes were mainly involved in sex pheromone sensing (HarmOR13 and HarmOR14b), reproduction (ejaculatory bulb-specific protein 3-like) and development (juvenile hormone esterase-like and forkhead box protein F2-like) (Table S3). These results indicate that the abnormal expression of *H. armigera*-derived alleles of sterile F_1_ hybrids is associated with the abnormal phenotypes of pheromone perception, sterility and developmental abnormalities [37]; the variance between the alleles from F_1_ hybrids and the genes expressed in parents and F_1_ hybrids is noticeable (Figure 2B), the possible reason for this was the removal of alleles that cannot clearly distinguish the alleles from *H. armigera* or *H. assulta* in F_1_ hybrids during the process of identifying the origin of alleles from F_1_ hybrids. In reciprocal crosses, the expression of alleles in F_1_ hybrids was species-specific (Figure 2D). The variance between groups is much greater than the variance within groups, and the variance between two parents is wide, which provides a basis for accurately predicting the parental origin of genes in F_1_ hybrids.

### 2.4. More Misexpressed Gene in the Antennae of Sterile and Reciprocal F_1_ Hybrids

**Figure 3:**
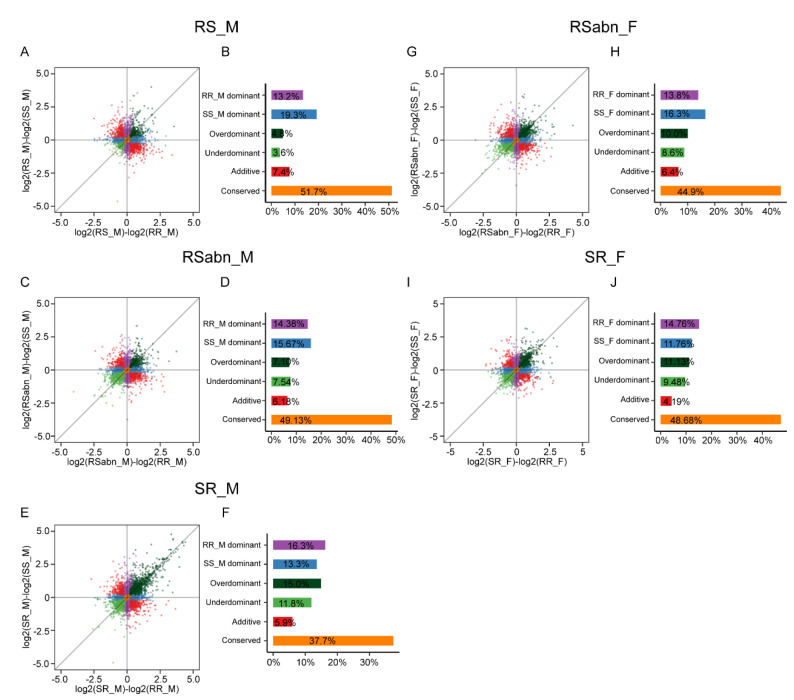
Inheritance modes of antennal gene expression in the five groups of F_1_ hybrids of *H. armigera* and *H. assulta*. Scatterplot in panels (**A**,**C**,**E**,**G**,**I**) illustrates the differences in expression level (log_2_(fold-change)) between F_1_ hybrids and each of the parental species. The *X*-axis represents the difference between RS_M and RR_M (**A**), RSabn_M and RR_M (**C**), SR_M and RR_M (**E**), RSabn_F and RR_F (**G**), and SR_F and RR_F (I); the *Y*-axis represents the difference between RS_M and SS_M (**A**), RSabn_M and SS_M (**C**), SR_M and SS_M (**E**), RSabn_F and SS_F (**G**), and SR_F and SS_F (**I**). Each point represents a single gene and is color-coded according to the inferred inheritance modes. Bar plots of panels (**B**,**D**,**F**,**H**,**J**) show the proportions of genes with different inheritance modes expressed in the antennae of RS_M (**B**), RSabn_M (**D**), SR_M (**F**), RSabn_F (**H**) and SR_F (**J**).

Following McManus et al. [38], we compared gene expression levels among *H. armigera*, *H. assulta* and F_1_ hybrids to infer the inheritance modes. The genes with low reads count in parental or allele genotypes were classified as uninformative, and the remaining genes were classified as follows: conserved, additive, RR-dominant, SS-dominant, underdominant, overdominant and ambiguous (Table S4). The underdominant or overdominant inheritance of gene expression refers to a gene expression level being either lower or higher in F_1_ hybrids than in any of the parental species, which is defined as a misexpression in F_1_ hybrids [39]. Among the five groups of F_1_ hybrids, RS_M had the lowest proportion of misexpressed genes. In initial F_1_ hybrids, the proportions of misexpressed genes in RSabn_M and RSabn_F were higher than those in RS_M (Figure 3A–D,G,H), indicating that RSabn_M and RSabn_F have abnormal gene expressions in antennae. A gene ontology (GO) enrichment analysis of misexpressed genes in RSabn_M and RSabn_F revealed that the genes were enriched in chemosensory-related classifications (e.g., olfactory receptor activity (GO:0004984)) (Tables S5 and S6), which explains why RSabn_M almost does not respond to pheromone components from the transcriptome (Figure 1B–D). It is worth noting that although both SR_M and SR_F were fertile, the proportions of misexpressed genes in SR_M and SR_F were much higher than those in RS_M, and approximately similar to or higher than those in RSabn_M and RSabn_F (Figure 3). This result demonstrates that the gene expression in antennae of SR_M and SR_F are also abnormal.

### 2.5. The Regulatory Patterns in Male Antennae Are Different from Those of Female Antennae

**Figure 4:**
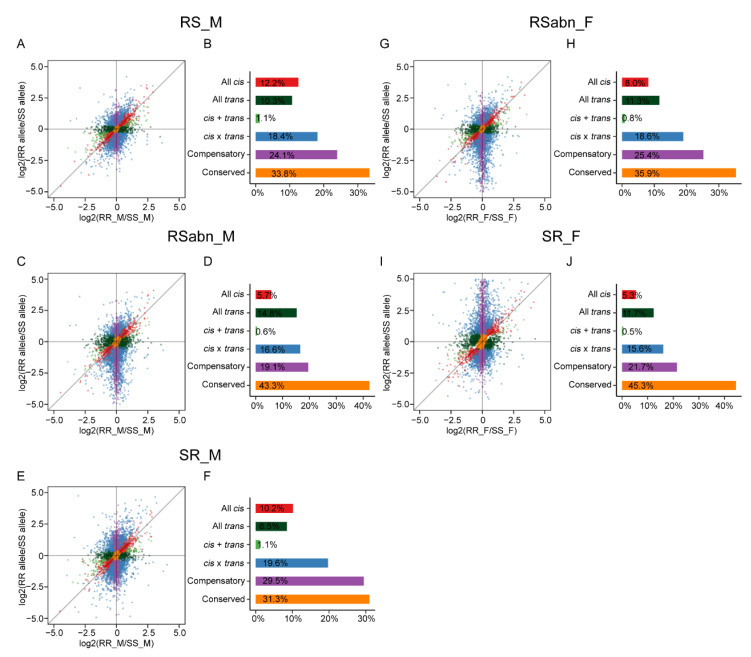
*cis*- and *trans*-regulatory variants of gene expression divergence in antennae of *H. armigera* and *H. assulta* was inferred by five groups of F_1_ hybrids. Scatterplots in panels (**A**,**C**,**E**,**G**,**I**) illustrate the differences in the expression between species versus between maternal and paternal alleles in F_1_ hybrids. The *X*-axis represents the gene expression divergence between RR_M and SS_M (**A**,**C**,**E**), RR_F and SS_F (**G**,**I**); the *Y*-axis represents the gene expression divergence between maternal and paternal alleles in RS_M (**A**), RSabn_M (**C**), SR_M (**E**), RSabn_F (**G**) and SR_F (**I**). Each point represents a single gene and is color-coded according to the inferred regulatory patterns. Bar plots of panels (**B**,**D**,**F**,**H**,**J**) show the proportions of genes with different regulatory patterns expressed in the antennae of RS_M (**B**), RSabn_M (**D**), SR_M (**F**), RSabn_F (**H**) and SR_F (**J**).

Antennae are among the most conspicuous, sexually dimorphic organs of insects [20,21]. We further investigated the role of *cis*- and *trans*-regulatory variants in the dimorphic differentiation of antennae. In cells of F_1_ hybrids, *cis*-regulatory elements have an allele-specific effect on gene expression, whereas *trans*-regulatory factors have an effect on the expression of both alleles [38,40,41]. Comparing the expression levels of the alleles of F_1_ hybrids with the expression levels of genes of the two parents can distinguish *cis*- and *trans*-regulatory variants between *H. armigera* and *H. assulta* (Figure S3). We first eliminated genes with low read counts in parental or allele genotypes as uninformative. The remaining genes were classified as conserved, all *cis*, all *trans*, *cis* + *trans*, *cis* × *trans*, compensatory and ambiguous (Table S7) based on the outcomes of statistical tests following the criteria outlined by Coolon et al. [42]. In the five groups of F_1_ hybrids, the proportions of genes classified as conserved were 31.3–45.3%, and those of compensatory were 19.1–29.5% (Figure 4), indicating that more than half of the genes that are classified into specific regulatory patterns have no difference in expression between the antennae of *H. armigera* and *H. assulta*. Among the genes that were divergently expressed between the two parents, we found that the regulatory patterns of gene expression divergence in the male antennae of *H. armigera* and *H. assulta* are different from those in female antennae. The proportion of genes classified as all *cis* was higher than that of all *trans* in both RS_M and SR_M, while the proportion of genes classified as all *trans* was higher than that of all *cis* in SR_F, and the proportion of genes classified as all *cis* in both RS_M and SR_M was higher than that of SR_F (Figure 4A,B,E,F,I,J and Figure S4D,E). These results indicate that the regulatory patterns of gene expression divergence in male antennae of *H. armigera* and *H. assulta* are different from those in female antennae, which could contribute to the differentiation of sexual dimorphism in antennae.

### 2.6. The Fertile Males in Initial F_1_ Hybrids Are More Reliable for Inferring Regulatory Patterns

The present results indicate that the regulatory patterns inferred from fertile F_1_ hybrids and sterile F_1_ hybrids were different. For example, the proportion of genes classified as all *cis* in RS_M was higher than that in RSabn_M, the proportion of genes classified as all *trans* in RS_M was lower than that in RSabn_M (Figure 4A–D and Figure S4A), and the proportion of genes classified as all *cis* in SR_F was lower than that in RSabn_F (Figure 4G–J and Figure S4B). Furthermore, we observed that the proportions of *H. assulta*-derived alleles in RSabn_M and RSabn_F were higher than those of *H. armigera*-derived alleles (Figure 4C,D,G,H), which can affect the inference of regulatory patterns. Unlike Bao, et al. [43], we observed that the crossing direction of hybridization had effects on the inference of regulatory divergence. Although the regulatory patterns inferred from RS_M were similar with those in SR_M, there were some differences (Figure 4A,B,E,F and Figure S4C). Although both RS_M and SR_M were fertile, SR_M was more difficult to obtain than RS_M by interspecific hybridization. Compared with RS_M, the other F_1_ hybrids expressed more misexpressed genes (Figure 3), which were strongly correlated with the conserved (Figure S5). These results show that these misexpressed genes are regulated by *trans*-factors resulting in the expression of both parental-derived alleles at similar levels in F_1_ hybrids. This further indicates that the signal regulatory network of these F_1_ hybrid has changed, which can affect the predicted results of regulatory patterns. Therefore, it is more accurate to use RS_M to infer regulatory patterns. This result provides a basis for accurately identifying regulatory patterns.

### 2.7. Contribution of cis- and trans-Regulatory Variants to Gene Expression Divergence in Antennae of H. armigera and H. assulta

**Figure 5:**
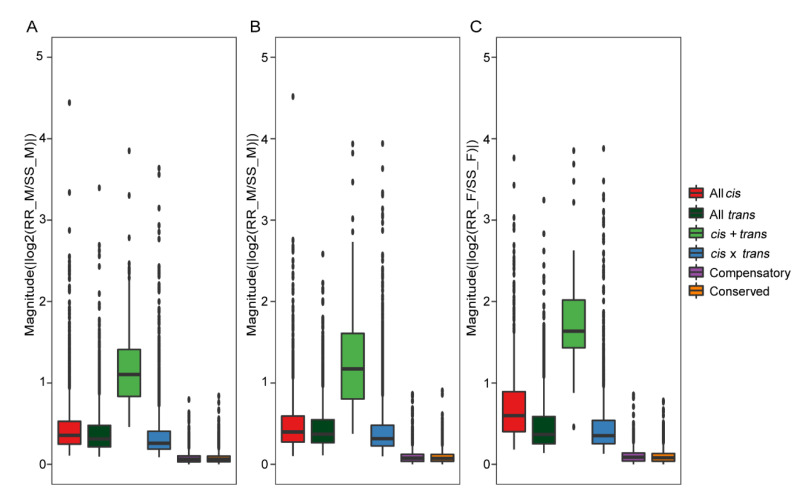
Expression divergence caused by regulatory patterns. (**A**–**C**) Boxplots showing the magnitude of expression divergence between species for genes classified as regulatory patterns in fertile F_1_ hybrids (RS_M (**A**), SR_M (**B**) and SR_F (**C**)), with regulatory patterns on the *X*-axis and a magnitude of expression divergence on the *Y*-axis. Differences in regulatory patterns were significant as indicated by Wilcoxon rank-sum tests.

To study the contribution of different regulatory patterns to gene expression divergence in antennae of *H. armigera* and *H. assulta*, we used the absolute value of log_2_(RR/SS) of fertile F_1_ hybrids to calculate the magnitude of gene expression divergence. In the fertile F_1_ hybrids, *cis* + *trans* regulation showed the highest level of gene expression divergence, followed by all *cis*, all *trans*, *cis* × *trans*, compensatory and conserved genes (*cis* + *trans* > all *cis* > all *trans* > *cis* × *trans* > compensatory and conserved; Wilcoxon rank-sum test, *p* < 0.05) (Figure 5 and Table S8). All *cis*-regulation made a stronger contribution to gene expression divergence in the antennae of two parents than all *trans*-regulation, and the contributions of *cis* × *trans* and compensatory were lower than those of all *cis* and all *trans*. It is worth noting that SR_F had higher levels of *cis* + *trans*, all *cis*, all *trans* and *cis* × *trans* regulatory divergences compared to RS_M and SR_M (Wilcoxon rank-sum test, *p* < 0.05) (Figure 5, Tables S9 and S10), suggesting that the difference between the male antennae of *H. armigera* and *H. assulta* is greater than that between the female antennae.

### 2.8. Regulatory Patterns of Olfactory-Related Protein Genes Expression in Antennae

Given that antennae are the most important olfactory organs of insects [21,44], we analyzed the regulatory patterns of olfactory-related protein genes expressed in the antennae, including ORs, odorant-binding proteins (OBPs), ionotropic receptors (IRs), and sensory neuron membrane proteins (SNMPs). Based on the above-mentioned analysis, we used RS_M to analyze the regulatory patterns of olfactory-related protein genes (File S1). We found that the proportions of genes classified as all *cis* were higher than those classified as all *trans* (Table S11), suggesting that *cis*-regulation plays a key role in the expression divergence of olfactory-related protein genes in antennae of *H. armigera* and *H. assulta*.

**Figure 6:**
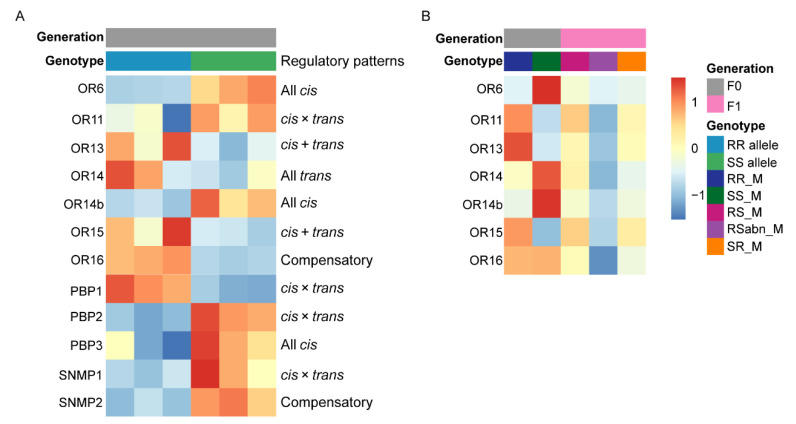
Expression patterns and regulatory patterns of pheromone perception related genes. (**A**) Heatmap displays the mean expression level of the ASE of pheromone receptor, pheromone binding protein and sensory neuron membrane protein genes for the three libraries in RS_M. Alleles of different origin are distinguished by different colors. The figure also shows the regulatory patterns of pheromone perception-related genes. (**B**) Heatmap displaying the pheromone receptor gene expression level for each genotype (RR_M, SS_M, RS_M, RSabn_M and SR_M) among the three libraries. Different parents and F_1_ hybrids are distinguished by different colors.

Because most of the olfactory sensilla on male antennae are related to sex pheromone sensing [23], we then analyzed the regulatory patterns of *PR* genes in male antennae, especially *OR13* and *OR14b*, the receptor genes of major sex pheromone components. In RS_M, the regulatory patterns of *OR13* and *OR14b* were *cis* + *trans* and all *cis*, respectively (Figure 6A). Both *HarmOR13* and *HassOR13* were expressed in RS_M antennae, and the most expressed gene was *HarmOR13*. Both *HarmOR14b* and *HassOR14b* were expressed in RS_M antennae, and the most expressed gene was *HassOR14b* (Figure 6A), indicating that the activity of the *cis*-regulatory elements of *HarmOR13* is stronger than that of *HassOR13*, and the activity of the *cis*-regulatory elements of *HassOR14b* is stronger than that of *HarmOR14b*. With regard to other *PR* genes, *OR6*, *OR11*, *OR15*, and *OR16*, and the genes associated with sex pheromone communication, pheromone binding proteins (*PBPs*) and *SNMPs* [45,46], were also mainly expressed as a parental allele in RS_M antennae (Figure 6A), and the regulatory patterns were also mainly *cis*-related regulations (Figure 6A). These results show that changes in *cis*-related regulations play an important role in the evolution of phenotypic differences in sex pheromones perception between male antennae of *H. armigera* and *H. assulta*.

We further analyzed the expression levels of *PR* in male antennae of RR_M, SS_M, RS_M, RSabn_M and SR_M, and found that RSabn_M has a low expression level of *PR* (Figure 6B), which explains that RSabn_M has almost no response to pheromone components (Figure 1B–D). Correlation analysis between the expression levels of *PR* genes and the EAG response of male antennae to Z11-16:Ald, Z9-16:Ald and Z9-14:Ald at 10 µg/µL found that *OR13*, *OR11* and *OR15* were closely related with Z11-16:Ald, and *OR16*, *OR14* and *OR14b* were closely related with Z9-16:Ald and Z9-14:Ald (Table S12), suggesting that these *PR* genes are critical for sex pheromone detection in *H. armigera* and *H. assulta*.

## 3. Discussion

In this research, we studied the role of *cis*- and *trans*-regulatory variants in gene expression divergence between antennae of *H. armigera* and *H. assulta*, and found that (1) regulatory patterns played different roles in gene expression divergence in antennae of males and females, (2) the regulatory patterns inferred by RS_M are more accurate, (3) *cis*-related regulations played a crucial role in the *PR* genes expression divergence in the antennae of *H. armigera* and *H. assulta*.

### 3.1. Genetic Basis of Gene Expression Divergence in Antennae of Related Insect Species

During the evolution of insects, the gene expression levels of *ORs* and other olfactory-related proteins in their antennae also changed to adapt to new environments [47]. However, the genetic basis of gene expression divergence in the antennae of related insect species is unknown. Previous research on the gene expression regulation of closely related insect species mainly focused on *Drosophila* and used the whole body or multiple tissues (e.g., head) [38,48]. Given that *cis*-regulatory elements usually drive expression within a single tissue [49], previous transcriptome data were actually collected from multiple tissues that could not capture the complete contribution of *cis*-regulatory changes. Studies of gene expression divergence in closely related species of *Drosophila* were often limited to females [9,38], which makes it impossible to study the genetic basis of the divergence of *PR* expression, because *PRs* are mainly expressed in male antennae [23,50]. Our previous studies showed that the hybridization of *H. armigera* and *H. assulta* can produce male and female F_1_ hybrids [18,19], which allowed us to study the genetic basis of expression divergence of genes, including *PRs*, in the two species and the regulatory divergence of male and female antennae.

### 3.2. Effects of cis- and trans-Regulatory Variants on Antennal Dimorphism

*cis*- and *trans*-regulatory variants are crucial to the development and evolution of sexual dimorphism [51,52]. *cis*-regulatory variants are characterized by low pleiotropy and low restriction, which are more beneficial or less harmful in the evolutionary process, are also more targeted than *trans*-regulatory variants [6,12,53,54,55]. However, *trans*-regulatory variants can affect the expression of genome-wide genes [56], are pleiotropic and more conducive to adaptive evolution, and result in greater variation [12]. Antennae are among the main organs that manifest phenotypic divergence in sexually dimorphic insects [20,21]. In this study, we found that the proportion of *cis*-regulatory genes was higher than the proportion of *trans*-regulatory genes in male antennae of *H. armigera* and *H. assulta*, whereas the trend was reversed in female antennae, suggesting that *cis*-regulatory variants and *trans*-regulatory variants play a key role in the gene expression divergence in male and female antennae, respectively. The principal components of *H. armigera* and *H. assulta* sex pheromones are identical, but the ratio is reversed [16]; therefore, a strong regulation of sex pheromone perception in males is required. Females of the two species need pleiotropic and strong adaptive changes to detect plant volatiles, as their host plants and plant volatiles are quite different [57,58]. In addition, we discovered that the magnitude of gene expression divergence in female antennae was higher than that of male antennae, which is conducive to the perception of different plant volatiles in females. Thus, we propose that *cis*- and *trans*-regulatory variants play a key role in the functional evolution of antennae of males and females in *H. armigera* and *H. assulta*. Our results make a clear connection between transcriptome data and phenotypic differences.

### 3.3. Regulatory Patterns Inferred by RS_M Is More Accurate

According to the Dobzhansky–Muller hybrid incompatibility theory [59,60], the sterility of F_1_ hybrid is caused by the incompatibility between the sites on the chromosomes of the F_1_ hybrid. The incompatibility of sites on chromosomes could lead to gene misexpression that affects regulatory patterns by causing cascade effects on downstream genes in regulatory networks [61,62]. Moreover, the misexpressed genes in sterile and relatively abnormal F_1_ hybrids are associated with tissue defects and developmental impairment [63]. These can affect the ASE of sterile F_1_ hybrids and thus affect the prediction of regulatory patterns. In our study, we found abnormal antennae development and gene expression in sterile F_1_ hybrids, and allele expression patterns were different between fertile and sterile F_1_ hybrids, which meant that regulatory patterns inferred from fertile F_1_ hybrids differed from sterile F_1_ hybrids. In addition, the regulatory patterns inferred from RS_M were different from SR_M. Although both RS_M and SR_M were fertile, misexpressed genes were frequent in SR_M than in RS_M. These misexpressed genes were strongly correlated with the conserved genes. This indicates that the signal regulatory network of these F_1_ hybrid has changed, which can affect the predicted results of regulatory patterns. Among the five groups of F_1_ hybrids, RS_M had the fewest misexpressed genes in the antennae. Therefore, it is relatively accurate to infer *cis*- and *trans*-regulatory variants from RS_M.

### 3.4. cis-Related Regulations Play a Crucial Role in Gene Expression Divergence of Pheromone Perception Related Protein Genes

The main function of PRs expressed in male antennae is detection of the sex pheromone released by females [22]. Differences in the expression levels and functions of PRs lead to changes in sex pheromone communication, which ultimately result in behavioral isolation of closely related species [27,50,64]. Therefore, studying the genetic basis of the expression divergence of *PRs* in males between related moths is important for understanding the mechanism of prezygotic isolation between closely related moth species. The types of PRs expressed in the antennae of *Heliothis*/*Helicoverpa* species are very similar. By altering the expression levels and functions of the PRs, the pheromone perception system in the male decodes the changes in the composition and ratio of sex pheromones released by the female [24,28,29,65,66]. Previous studies have shown that *cis*- and *trans*-regulatory variants participate in the regulation of the expression divergence of olfactory-related protein genes in related insects [67]. In *H. armigera* and *H. assulta* males, the expression levels of the two major *PR* genes, *OR13* and *OR14b*, are contrasting [28,29]. In this study, we found that the regulatory patterns of *OR13* and *OR14b* were *cis* + *trans* and all *cis*, respectively. Most allele-specific variations are controlled by *cis*-regulatory elements located near genes [68,69]. In RS_M, *OR13* was mainly derived from *H. armigera*, and *OR14b* was mainly derived from *H. assulta*. Other *PRs,* as well as *PBPs* and *SNMPs,* were mainly expressed as a parental allele. These findings indicate that *cis*-related regulations are important in the evolution of sex pheromones in the perception of *H. armigera* and *H. assulta*. In general, *cis*-related regulations are less limited and have larger effects on gene expression than *trans*-related regulations, achieving a more precise and rapid regulation of the expression of *PR* genes [6,12,53,54,70]. Sex pheromone communication is very critical in the reproduction of moth species [71], and the changes in *cis*-related regulations of *PR* genes expression are conducive to the quick and effective adaption of males to the changes in sex pheromone production in females. Our results also show that the ASE analysis of hybrids is quite powerful for understanding how specific changes in PR gene expression can contribute to rapid evolutionary changes in sex pheromones perception.

### 3.5. Relationships between the Expression Levels of PRs and the Electrophysiological Activities of Antennae

Correlation analyses showed that the expression levels *OR13*, *OR11* and *OR15* were strongly correlated with the EAG responses to Z11-16:Ald. OR13, tuned to the major pheromone component Z11-16:Ald, is expressed in one OSN of the A type sensilla of male antennae, while OR11 is expressed in another OSN of the same sensilla [72]. Previous studies showed that the ORs of moths with close evolutionary relationships may have gene duplications or be linked on chromosomes [65]. *OR15* is phylogenetically clustered in a clade with *OR11* and *OR13* [66], and perhaps closely linked with *OR13* or *OR11* on the same chromosome. The correlation analyses also showed that the expression levels of *OR16*, *OR14*, and *OR14b* are strongly correlated with the EAG responses to Z9-16:Ald and Z9-14:Ald. Previous studies showed that OR14b was tuned to Z9-16:Ald in *H. assulta*, but tuned to Z9-14:Ald in *H. armigera*; OR16 was also tuned to Z9-14:Ald in both species [28,66]. *OR14b* is expressed in one OSN of the C type sensilla, and *OR16* is expressed in another OSN of some C type sensilla [28]. *OR14* is phylogenetically clustered in a clade with *OR14b* [66], perhaps closely linked with *OR14b* on the same chromosome. Therefore, the correlation analysis conducted in the present study to predict the characteristics of PRs and their roles in sex pheromone perception is informative and credible.

## 4. Materials and Methods

### 4.1. Insect Rearing and Interspecific Hybridization

The larvae of *H. armigera* and *H. assulta* were collected in tobacco (*Nicotiana tabacum*) fields in Luoyang, Henan Province, China, and reared under a 16L:8D photoperiod cycle at 26 ± 1 °C and 55–65% relative humidity in the laboratory. Given that both species are agricultural pests in China, no special permission was required for collection and experimentation. The larvae of *H. armigera*, *H. assulta* and their F_1_ hybrids were reared on the same artificial diet, the main component of which was wheat germ [73]. Adults were fed with 10% honey water.

In the pupal stage, the females and males were separated and placed in separate cages for emergence. After emergence, 30 pairs of female *H. armigera* and male *H. assulta*, and 30 pairs of female *H. assulta* and male *H. armigera,* were placed in cylindrical mating cages (diameter 30 cm and height 35 cm) to obtain F_1_ hybrids. To improve the success rate of interspecific hybridization and the survival rate of F_1_ hybrids eggs, the number of parental individuals was increased and a conical flask containing a bunch of fresh tobacco leaves in was placed in the cages. Eggs were collected daily and F_1_ hybrids larvae were fed on the artificial diets.

### 4.2. Antennae Collection, RNA Extraction and Transcriptome Sequencing

Regarding the antennae of adults, 3–4 days after emergence from nine groups of insects, RR_M, SS_M, RS_M, RSabn_M, SR_M, RR_F, SS_F, RSabn_F and SR_F were separately collected and immediately frozen in liquid nitrogen. The samples were stored at –80 °C until use. Thirty individuals were used in each group of insects for each replicate, and three replicates were run, except in SR_F. Given the limited number of SR_F, only two replicates were set.

Total RNA was extracted from the antennae of each group of samples with the RNeasy Plus Universal Mini Kit (QIAGEN, Hilden, Germany) following the manufacturer’s instructions. A total amount of 1.5 µg RNA per sample was used for transcriptome sequencing. Sequencing libraries were prepared using the NEBNext^®^ Ultra™ RNA Library Prep Kit for Illumina. The library was sequenced on the Illumina HiSeq4000 platform of Allwegene Co., Ltd. (Beijing, China). The PE150 strategy was used to generate the paired-end reads.

### 4.3. Processing of RNA-seq Datasets

After sequencing, 26 RNA-seq libraries, the raw reads were filtered using Trimmomatic v0.33 [74] to remove reads with sequencing adapters, N content greater than 10%, and low-quality base (Q ≤ 20) content greater than 50% reads. A total of 1.28 billion clean reads were obtained. Clean reads were analyzed with FastQC v0.11.9 (https://www.bioinformatics.babraham.ac.uk/projects/fastqc/ (accessed on 1 August 2019)) to examine their quality. Given the high degree of variation and divergence of sequences among the samples, we used the variation-tolerant aligner Stampy v1.0.31 [75] for reads mapping with all parameters set to default values. Filtered RNA-seq reads were processed by HyLiTE v2.0.2 for ASE [36], which required mapping all reads to a single reference transcriptome or genome. We used transcript sequences of the genes annotated from the *H. armigera* genome as the reference [76]. Owing to the lack of *OR13* and *OR14b*, we assembled 28 transcriptome samples sequenced in the *H. armigera* genome article to re-annotate.

### 4.4. Measuring ASE and PCA Analysis

As explained earlier, we used Stampy v1.0.31 to align reads [75]. Stampy generates a sam file that maps each sample to the reference transcriptome. We separated the parents and F_1_ hybrids according to sex, and analyzed the reads with HyLiTE separately for each sex [36]. We called HyLiTE with the following command: ‘HyLiTE -v -S -f sam_protocol_file_Female/Male.txt -r Harm_1.0_rna.fasta -n my_first_Female/Male_HyLiTE’. The main output of HyLiTE includes the reads count file of each allele in each F_1_ hybrid sample and the expression file of each gene in each F_1_ hybrid sample and the two parents. According to the presence and absence of diagnostic parental SNPs, reads were divided into three categories: from one parent, from two parents and unknown [36]. The percentages of allelic reads of the F_1_ hybrids of *H. armigera* and *H. assulta* are summarized in the Tables S1 and S2.

After performing the HyLiTE analysis, we performed variance stability transformation on the reads count data to remove the experiment-wide trend and cluster samples into interesting groups [77]. PCA was performed using the prcomp package of R v4.1.2.

### 4.5. Classification of Inheritance Modes

Following McManus et al. [38], the expression levels of the F_1_ hybrids and the two parents were compared for each gene in three comparisons: (1) the expression of the gene in *H. armigera* versus in *H. assulta*, (2) the expression of the gene in *H. armigera* versus in F_1_ hybrids, and (3) the expression of the gene in *H. assulta* versus in F_1_ hybrids. Based on the research of Wang et al. [77], DESeq2 v1.32.0 was used for normalization, differential expression tests, and classification of inheritance modes. The genes were classified into the following inheritance modes: conserved, additive, RR-dominant, SS-dominant, underdominant, overdominant and ambiguous.

### 4.6. Classification of Regulatory Patterns

*cis*- and *trans*-regulatory variants were analyzed by combining the parental gene expression and the allele expression level of the F_1_ hybrid. After the RNA-seq data were processed with HyLiTE, we directly determined the relative gene expression level of the parents and hybrid alleles, and then analyzed the *cis*- and *trans*-regulatory variants. The criteria of McManus et al. [38] were followed in that three comparisons were needed to classify gene regulation: (1) the difference in parental genotype expression (*H. armigera* gene/*H. assulta* gene), (2) the difference in F_1_ hybrid allele expression (*H. armigera* allele/*H. assulta* allele), and (3) the ratio of parental genotype expression level difference and F_1_ hybrid allele expression difference (*H. armigera* gene/*H. assulta* gene)/ (*H. armigera* allele/*H. assulta* allele). Following the research of Wang et al. [77], DESeq2 v1.32.0 was used for normalization, differential expression tests, and classification of regulatory patterns. We eliminated from consideration genes with low read counts in parental or allele genotypes as uninformative. The remaining genes were classified as conserved, all *cis*, all *trans*, *cis* + *trans*, *cis* × *trans*, compensatory and ambiguous. To compare the contribution of *cis*- and *trans*-regulatory variants to the antennal gene expression divergence between *H. armigera* and *H. assulta*, the log_2_-transformed fold changes were first tested for normality using the Shapiro–Wilk test, followed by Wilcoxon rank-sum tests for the data that were not normally distributed [77]. All test statistics were analyzed in R v4.1.2.

### 4.7. Functional Annotation and Gene Ontology

The annotation of olfactory-related genes annotation was based on the *H. armigera* genome annotation [76] and comprised 77 *ORs*, 28 *IRs*, 42 *OBPs* and 2 *SNMPs*. The heatmaps of *PR* genes expression levels in antennae were plotted using the R package ‘pheatmap’ [78].

The gene UniProt ID was obtained by performing DIAMOND blastx search against the Swiss-Prot database [79]. The GO term of the corresponding gene was retrieved through id-mapping with the Swiss-Prot database. The R package ‘clusterProfiler’ was used to perform GO enrichment analysis of the different gene sets [80], with an adjusted *p* value cutoff of 0.05.

### 4.8. DNA Extraction and Genomic PCR

We used the MiniBEST Universal Genomic DNA Extraction Kit Ver.5.0 (TaKaRa) to extract genomic DNA from pupae. The purity and concentration of the DNA was detected using Nano Drop 2000 spectrophotometer (Thermo Scientific, Wilmington, DE, USA). The purified genomic DNA was stored at −20 °C.

We designed a pair of primers to amplify *GOBP1*, *TP1* and *GUW1* (Table S13). PCR amplifications were conducted in a 25 µL reaction volume with Premix TaqTM Ver.2.0 (TaKaRa) using a thermal cycler. The thermal cycling conditions were set as follows: 98 °C for 2 min; then 35 cycles of 98 °C for 10 s, 55 °C for 30 s, and 72 °C for 1 min; and 72 °C for 10 min. The PCR products were analyzed on 1.2% agarose gels.

### 4.9. EAG Recordings

Solutions of (*Z*)-11-hexadecenal (Z11-16:Ald), (*Z*)-9-hexadecenal (Z9-16:Ald) and (*Z*)-9-tetradecenal (Z9-14:Ald) (Table S14) were prepared in the solvent (paraffin oil) (Sigma) at different concentrations (0.01, 0.1, 1, 10, 100 μg/μL). Paraffin oil was used as the control. EAG experiments were performed, and EAG values were recorded according to the method of Zhao et al. [37]. The EAG signals were recorded with EAG-adapted software (Syntech, Hilversum, The Netherlands).

### 4.10. Correlation Analysis

Spearman correlation analysis was conducted between the *PR* expression levels of male antennae (RR_M, SS_M, RS_M, RSabn_M and SR_M) and the EAG values of the male antennae to 10 µg/µL Z11-16:Ald, Z9-16:Ald and Z9-14:Ald.

## 5. Conclusions

We explored the regulatory patterns of the gene expression divergence of antennae in closely related insects. We observed that the regulatory patterns of gene expression divergence in male antennae were different from those in females; the contribution of regulatory patterns to gene expression divergence in males was less than that in females; and *cis*-related regulations played a crucial role in the evolution of sex pheromone perception in moths. This research is helpful for understanding the mechanisms of the regulatory patterns in the expression divergence of olfactory-related genes for the antennae of *H. armigera* and *H. assulta*, especially in *PRs*. Furthermore, a comprehensive annotation of the genomes of *H. armigera* and *H. assulta*, together with ATAC-seq and ChIP-seq, would greatly contribute to the elucidation of the specific mechanisms of the *cis*- and *trans*-regulatory variants on *PRs* in *H. armigera* and *H. assulta*, especially *OR13* and *OR14b*.

## Data Availability

The data presented in this study are available on request from the corresponding author on reasonable request.

## Supplementary Materials

The supporting information can be downloaded at: https://www.mdpi.com/article/10.3390/ijms231710050/s1.
