# Supplementary material for: The Genetic Basis of Gene Expression Divergence in Antennae of Two Closely Related Moth Species, Helicoverpa armigera and Helicoverpa assulta
Source: Int J Mol Sci. 2022 Sep 2;23(17):10050. doi: 10.3390/ijms231710050 (PMC9456569; doi:10.3390/ijms231710050)
Supplement: Supplementary file 1 [file ijms-23-10050-s001.zip › Supplementary Tables and Figures.pdf]

Supplementary Tables and Figures

Table S1. Mapping of RNA-seq reads from male F_1 hybrids to *H. armigera*.

	Total or average
Total number of reads mapping on the reference	349900334
Number of reads unambiguously assigned to a parent	188125299
Proportion of reads unambiguously assigned to parent	53.77%
Number of reads unambiguously assigned to RR	91596438
Number of reads unambiguously assigned to SS	96528861
Number of reads with uninformative assignment	51191609
Number of reads with unknown or ambiguous assignment	110583426
Total number of SNPs identified	4217188
Total number of child unique SNPs	2253773

Note: This table presents the number of reads generated *vs* those that could be unambiguously mapped to one parental genome or the other based on species-informative single nucleotide variants using Stampy (see Materials and methods). The right-most column reports the total of RS_M, RSabn_M and SR_M. RR = *H. armigera,* SS = *H. assulta*.

Table S2. Mapping of RNA-seq reads from female F_1 hybrids to *H. armigera*.

	Total or average
Total number of reads mapping on the reference	184292437
Number of reads unambiguously assigned to a parent	99587705
Proportion of reads unambiguously assigned to parent	54.04%
Number of reads unambiguously assigned to RR	49860105
Number of reads unambiguously assigned to SS	49727600
Number of reads with uninformative assignment	27985208
Number of reads with unknown or ambiguous assignment	56719524
Total number of SNPs identified	3826144
Total number of child unique SNPs	1909584

Note: This table presents the number of reads generated *vs* those that could be unambiguously mapped to one parental genome or the other based on species-informative single nucleotide variants using Stampy (see Materials and methods). The right-most column reports the total of RSabn_F and SR_F. RR = *H. armigera,* SS = *H. assulta*.

Table S3. Annotation of *H. armigera*-derived alleles expressed in RSabn_M and RSabn_F.

Gene ID	Loading scores	Gene names
XM_021330508.1	0.076079	HarmOR14
HarmOR14b	0.074236	HarmOR14b
XM_021333189.1	0.072903	uncharacterized LOC110375164 (LOC110375164), mRNA
XM_021345446.1	0.068927	cytochrome P450 6B6 (LOC110384248), mRNA
XM_021329886.1	0.068539	multidrug resistance protein 1B-like (LOC110372874), mRNA
HarmOR13	0.062859	HarmOR13
XM_021330489.1	0.061132	odorant receptor 13a-like (LOC110373267), transcript variant X1, mRNA
XM_021338031.1	0.060224	mucin-3A (LOC110378669), mRNA
XM_021334791.1	0.059157	ejaculatory bulb-specific protein 3-like (LOC110376344), mRNA
XM_021326364.1	0.058879	cytochrome P450 4c3-like (LOC110370525), mRNA
XM_021336226.1	0.058256	juvenile hormone esterase-like (LOC110377360), mRNA
XM_021330490.1	0.056229	odorant receptor 13a-like (LOC110373267), transcript variant X2, mRNA
XM_021343539.1	0.054088	probable cytochrome P450 6a13 (LOC110382836), mRNA
XM_021327841.1	0.053254	uncharacterized protein K02A2.6-like (LOC110371520), mRNA
XM_021328832.1	0.049859	pollen-specific leucine-rich repeat extensin-like protein 1 (LOC110372241), mRNA
XM_021329235.1	0.04939	forkhead box protein F2-like (LOC110372486), transcript variant X1, mRNA
XR_002429515.1	0.049336	uncharacterized LOC110376029 (LOC110376029), ncRNA
XM_021329693.1	0.048717	uncharacterized LOC110372738 (LOC110372738), mRNA
XM_021331577.1	0.048661	acyl-CoA synthetase short-chain family member 3, mitochondrial (LOC110374040),
XM_021329237.1	0.048315	forkhead box protein F2-like (LOC110372486), transcript variant X2, mRNA

Note: Annotation of the top 20 genes that contributed to the variance of the *H. armigera*-derived alleles expressed in RSabn_M and RSabn_F.

Table S4. Formal criteria for classifying inheritance modes. RR = *H. armigera*, SS = *H. assulta*.

	RR vs SS	Hybrid vs SS	Hybrid vs RR	Classification
1	RR = SS	Hybrid = SS	Hybrid = RR	conserved
2	RR < SS	Hybrid < SS	Hybrid > RR	additive
3	RR > SS	Hybrid > SS	Hybrid < RR	additive
4	RR < SS	Hybrid < SS	Hybrid = RR	RR dominant
5	RR > SS	Hybrid > SS	Hybrid = SS	RR dominant
6	RR < SS	Hybrid = SS	Hybrid > RR	SS dominant
7	RR > SS	Hybrid = SS	Hybrid < RR	SS dominant
8	RR = SS	Hybrid < SS	Hybrid < RR	underdominant
9	RR < SS	Hybrid < SS	Hybrid < RR	underdominant
10	RR > SS	Hybrid < SS	Hybrid < RR	underdominant
11	RR = SS	Hybrid > SS	Hybrid > RR	overdominant
12	RR < SS	Hybrid > SS	Hybrid > RR	overdominant
13	RR > SS	Hybrid > SS	Hybrid > RR	overdominant
14	RR = SS	Hybrid = SS	Hybrid ≠ RR	ambiguous
15	RR = SS	Hybrid ≠ SS	Hybrid = RR	ambiguous
16	RR = SS	Hybrid < SS	Hybrid > RR	ambiguous
17	RR = SS	Hybrid > SS	Hybrid < RR	ambiguous
18	RR ≠ SS	Hybrid = SS	Hybrid = RR	ambiguous
19	RR < SS	Hybrid = SS	Hybrid < RR	ambiguous
20	RR > SS	Hybrid = SS	Hybrid > RR	ambiguous
21	RR < SS	Hybrid > SS	Hybrid = RR	ambiguous
22	RR > SS	Hybrid < SS	Hybrid = RR	ambiguous
23	RR < SS	Hybrid > SS	Hybrid < RR	ambiguous
24	RR > SS	Hybrid < SS	Hybrid > RR	ambiguous

Table S5. GO enrichment analysis of misexpressed genes in RSabn_M antennae.

ID	Description	*P* value	Counts
**GO:0005549**	**odorant binding**	**1.78E-11**	**23**
**GO:0004984**	**olfactory receptor activity**	**1.20E-09**	**21**
**GO:0050911**	**detection of chemical stimulus involved in sensory perception of smell**	**5.14E-07**	**18**
GO:0002181	cytoplasmic translation	2.49E-06	15
GO:0045599	negative regulation of fat cell differentiation	1.78E-05	7
GO:0022627	cytosolic small ribosomal subunit	3.01E-05	11
GO:0050796	regulation of insulin secretion	3.60E-05	8
GO:0034185	apolipoprotein binding	4.43E-05	7
GO:0015171	amino acid transmembrane transporter activity	5.60E-05	14
GO:0004129	cytochrome-c oxidase activity	9.61E-05	7
GO:0150094	amyloid-beta clearance by cellular catabolic process	9.61E-05	7
GO:0032590	dendrite membrane	9.83E-05	13
GO:0003735	structural constituent of ribosome	0.000115	34
GO:0008574	ATP-dependent microtubule motor activity	0.000188	7
GO:0022626	cytosolic ribosome	0.000207	9
GO:0016600	flotillin complex	0.000258	6
GO:0033391	chromatoid body	0.000258	6
GO:0061351	neural precursor cell proliferation	0.000258	6
GO:0070822	Sin3-type complex	0.000258	6
GO:0022625	cytosolic large ribosomal subunit	0.000268	10
GO:0071392	cellular response to estradiol stimulus	0.000341	7
GO:0005865	striated muscle thin filament	0.000415	13

Table S6. GO enrichment analysis of misexpressed genes in RSabn_F antennae.

ID	Description	*P* value	Counts
GO:0022625	cytosolic large ribosomal subunit	9.02E-05	12
GO:0048787	presynaptic active zone membrane	9.31E-05	7
GO:0043195	terminal bouton	0.000139	30
GO:0036098	male germ-line stem cell population maintenance	0.00019	9
**GO:0004984**	**olfactory receptor activity**	**0.000207**	**16**
GO:0051721	protein phosphatase 2A binding	0.000216	12
GO:0016281	eukaryotic translation initiation factor 4F complex	0.000225	7
GO:0140326	ATPase-coupled intramembrane lipid transporter activity	0.000225	7
GO:0022626	cytosolic ribosome	0.000256	10
GO:0022627	cytosolic small ribosomal subunit	0.000299	11
GO:0043186	P granule	0.000301	15
GO:0045087	innate immune response	0.0004	39
GO:0046529	imaginal disc fusion	0.000412	8
GO:0008239	dipeptidyl-peptidase activity	0.000453	11
GO:1902017	regulation of cilium assembly	0.000453	11
GO:0017146	NMDA selective glutamate receptor complex	0.000473	7
GO:0007281	germ cell development	0.000496	18
GO:0030018	Z disc	0.000532	31
GO:0007269	neurotransmitter secretion	0.00065	29
**GO:0005549**	**odorant binding**	**0.000718**	**15**
GO:0009792	embryo development ending in birth or egg hatching	0.000718	15
GO:0004890	GABA-A receptor activity	0.000722	8
GO:0031629	synaptic vesicle fusion to presynaptic active zone membrane	0.000722	8
GO:0042023	DNA endoreduplication	0.000722	8
GO:0007297	ovarian follicle cell migration	0.000899	7
**GO:0050911**	**detection of chemical stimulus involved in sensory perception of smell**	**0.000937**	**15**
GO:0005112	Notch binding	0.00095	10
GO:0045751	negative regulation of Toll signaling pathway	0.001021	6
GO:1990904	ribonucleoprotein complex	0.001153	30
GO:0042438	melanin biosynthetic process	0.001195	8
GO:0001822	kidney development	0.001269	12
GO:0030833	regulation of actin filament polymerization	0.001269	12
GO:0033674	positive regulation of kinase activity	0.001269	12
GO:1903688	positive regulation of border follicle cell migration	0.001341	16
GO:0005319	lipid transporter activity	0.001348	11

Table S7. Formal criteria for classifying *cis* and *trans* genetic contributions. RR = *H. armigera,* SS = *H. assulta*.

	RR vs SS	RR allele vs SS allele	TransTest	Classification
1	RR = SS	RR allele = SS allele	RR/SS = RR allele/SS allele	Conserved
2	RR = SS	RR allele ≠ SS allele	RR/SS ≠ RR allele/SS allele	Compensatory
3	RR ≠ SS	RR allele = SS allele	RR/SS ≠ RR allele/SS allele	All *trans*
4	RR ≠ SS	RR allele ≠ SS allele	RR/SS = RR allele/SS allele	All *cis*
5	RR ≠ SS	RR allele ≠ SS allele	RR/SS ≠ RR allele/SS allele	*cis* and *trans*
5.1	RR ≠ SS	RR allele ≠ SS allele	RR/SS > RR allele/SS allele	*cis* + *trans*
5.2	RR ≠ SS	RR allele ≠ SS allele	RR/SS < RR allele/SS allele	*cis* × *trans*

Table S8. Statistical analysis of the contribution of various regulatory patterns in five groups of F_1 hybrids. Statistical significance of the difference between different regulatory patterns was determined with Wilcoxon rank-sum test.

Groups	Difference comparison	*P* value
RS_M	*cis* + *trans* and all *cis*	< 2.2E-16
	all *cis* and all *trans*	2.27E-10
	all *trans* and *cis* × *trans*	4.05E-16
	cis × *trans* and compensatory	2.20E-16
	compensatory and conserved	0.6798
SR_M	*cis* + *trans* and all *cis*	< 2.2E-16
	all *cis* and all *trans*	5.28E-03
	all *trans* and *cis* × *trans*	3.44E-14
	cis × *trans* and compensatory	< 2.2E-16
	compensatory and conserved	0.4396
SR_F	*cis* + *trans* and all *cis*	< 2.2E-16
	all *cis* and all *trans*	< 2.2E-16
	all *trans* and *cis* × *trans*	0.1404
	cis × *trans* and compensatory	< 2.2E-16
	compensatory and conserved	0.001295

Table S9. Analysis of contribution of predicted regulatory patterns to gene expression divergence by RS_M and SR_F. Statistical significance of the difference between different regulatory patterns was determined with Wilcoxon rank-sum test.

Regulatory patterns	Median (RS_M)	Median (SR_F)	Difference comparison	*P* value
cis + *trans*	1.104826	1.670548	*cis* + *trans* (RS_M) and *cis* + *trans* (SR_F)	2.86E-12
All *cis*	0.3629841	0.6085473	all *cis* (RS_M) and all *cis* (SR_F)	< 2.2E-16
All *trans*	0.3194907	0.3708606	all *trans* (RS_M) and all *trans* (SR_F)	3.48E-16
cis × *trans*	0.2622207	0.3618743	*cis* × *trans* (RS_M) and *cis* × *trans* (SR_F)	< 2.2E-16

Table S10. Analysis of contribution of predicted regulatory patterns to gene expression divergence by SR_M and SR_F. Statistical significance of the difference between F_1 hybrids of different sex was determined with Wilcoxon rank-sum test.

Regulatory patterns	Median (SR_M)	Median (SR_F)	Difference comparison	*P* value
cis + *trans*	1.203098	1.670548	*cis* + *trans* (SR_M) and *cis* + *trans* (SR_F)	2.38E-08
All *cis*	0.3982799	0.6085473	all *cis* (SR_M) and all *cis* (SR_F)	< 2.2E-16
All *trans*	0.3644779	0.3708606	all *trans* (SR_M) and all *trans* (SR_F)	0.1073
cis × *trans*	0.310307	0.3618743	*cis* × *trans* (SR_M) and *cis* × *trans* (SR_F)	< 2.2E-16

Supplementary Table S11. Regulatory patterns of olfactory-related genes.

Gene type	*ORs*	*OBPs*	*IRs*	*SNMPs*
Number of genes	77	42	28	2
All *cis*	14.3%	9.5%	14.3%	0%
All *trans*	9.1%	7.1%	0%	0%
cis + *trans*	3.9%	0%	0%	0%
cis × *trans*	18.2%	23.8%	21.4 %	50%
Compensatory	14.3%	16.7%	14.3%	50%
Conserved	6.5%	11.9%	7.1%	0%
Ambiguous	19.5%	4.8%	17.9%	0%
Uninformative	14.3%	26.2%	25%	0%

Note: *ORs*: odorant receptors; *OBPs*: odorant-binding proteins; *IRs*: ionotropic receptors; *SNMPs*: sensory neuron membrane proteins.

Table S12. Correlation coefficients between the gene expression levels of PRs in antennae and electrophysiological response.

	OR6	*OR11*	*OR13*	*OR14*	*OR14b*	*OR15*	*OR16*
Z11-16:Ald	-0.4 (0.52)	0.9 (0.083)	0.9 (0.083)	0.1 (0.95)	0 (1)	0.8 (0.13)	0.3 (0.68)
Z9-16:Ald	0.3 (0.68)	0.3 (0.68)	0.3 (0.68)	0.7 (0.23)	0.6 (0.35)	-0.1 (0.95)	0.9 (0.083)
Z9-14:Ald	0.4 (0.52)	0.4 (0.52)	0.4 (0.52)	0.9 (0.083)	0.7 (0.23)	0 (1)	1 (0.017)

Note: The table shows the correlation analysis between the electrophysiological responses of the antennae in RR_M, SS_M, RS_M, RSabn_M and SR_M to 10 μg/μL of Z11-16:Ald, Z9-16:Ald and Z9-14:Ald and the gene expression levels of *PRs*. The corresponding *P*-values are in parentheses.

Table S13. Primers used for RT-PCR of autosomal marker gene *GOBP1*, Z chromosome marker gene *TPI*, W chromosome marker gene *GUW1*.

Gene	Primer	Sequence (5’ - 3’)
GOBP1	F	CACCGAGAAGTTCATCCAGTCG
	R	CTTGAAGCACTCGGCCACGTGC
TPI	F	TGTTGCCGCACAAAACTG
	R	CTTACTTTCTCTGCAAC
GUW1	F	CAATAGACGGCGTGATGTTT
	R	ATCGCTTCGCCTCTTTAACT

Note: F: forward strand; R: reverse strand.

Table S14. Tested sex pheromone compounds for functional analysis of RR_M, SS_M, RS_M, RSabn_M and SR_M.

Compounds	Purity	Supplier
Z11-16:Ald	95%	Shin-Etsu
Z9-16:Ald	95%	Shin-Etsu
Z9-14:Ald	95%	Shin-Etsu

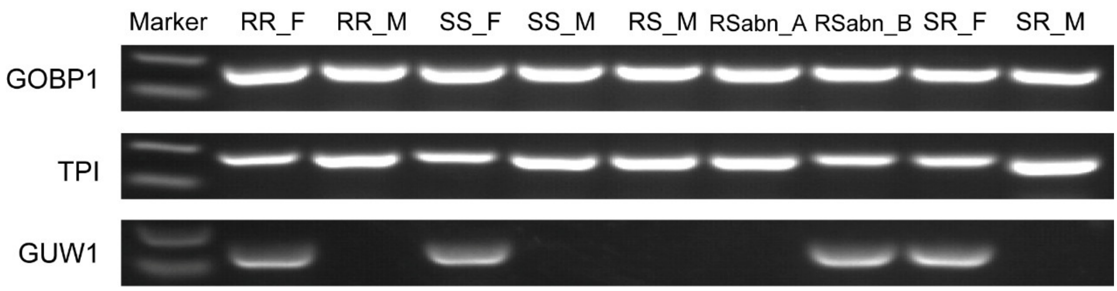

Figure S1. Identification of the sex of the two sterile abnormal F_1 hybrids. The figure shows the expression of the autosomal marker gene *GOBP1*, Z chromosome marker gene *TPI* and W chromosome marker gene *GUW1* of RR_F, RR_M, SS_F, SS_M, RS_M, RSabn_M, SR_M, RSabn_F and SR_F as amplified by PCR.

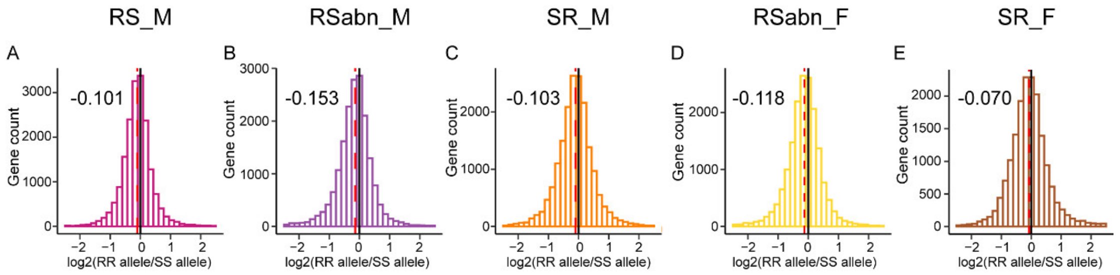

Figure S2. Histograms of allele-specific expression direction and magnitude of F_1 hybrids. Median $\log_2$ ratios of RR allele/SS allele expression is taken for each gene in F_1 hybrids (RS_M (A), RSabn_M (B), SR_M (C), RSabn_F (D) and SR_F (E)). The black solid line at zero (x = 0) indicates the same expression of the RR allele and SS allele, and the red dotted line indicates the median of the allele-specific expression bias observed in the antennae of F_1 hybrids.

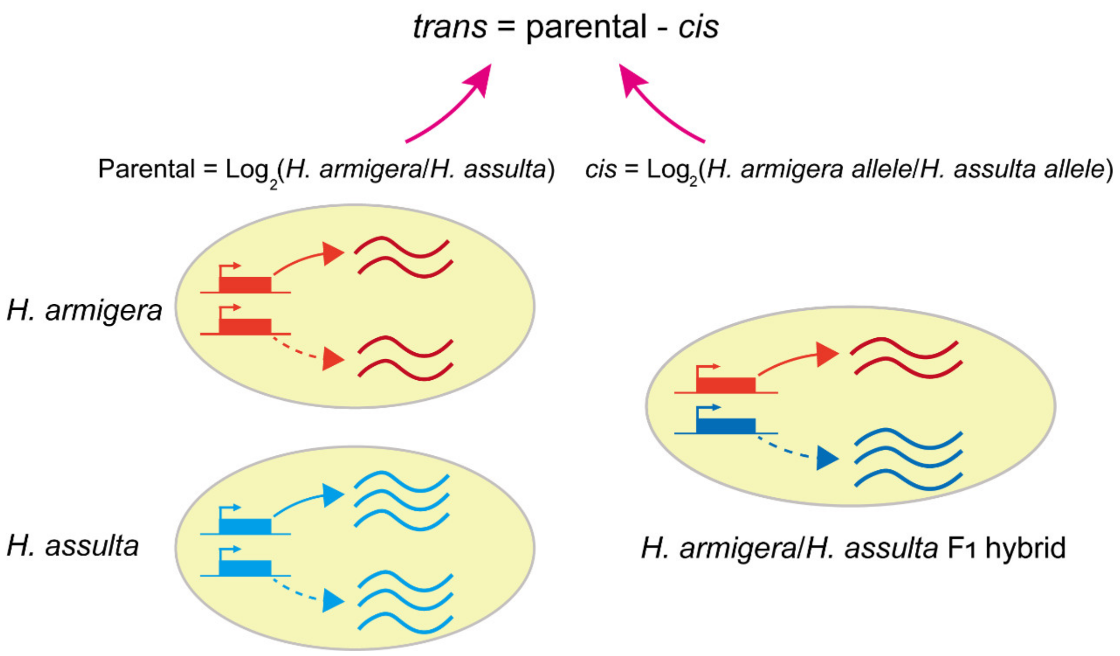

Figure S3. Estimation of *cis*- and *trans*-regulatory variants from the difference between the antennal gene expression of parents and F_1 hybrid allele-specific expression. The difference in antennal gene expression between *H. armigera* and *H. assulta* reflects both *cis*- and *trans*-regulatory variants, whereas the expression of the same gene in the common *trans*-regulatory environment of the F_1 hybrid reflects *cis*-regulatory variants between *H. armigera* and *H. assulta* alleles. Although *trans*-regulatory variants could not be directly measured, they could be inferred by the difference in parental-*cis*.

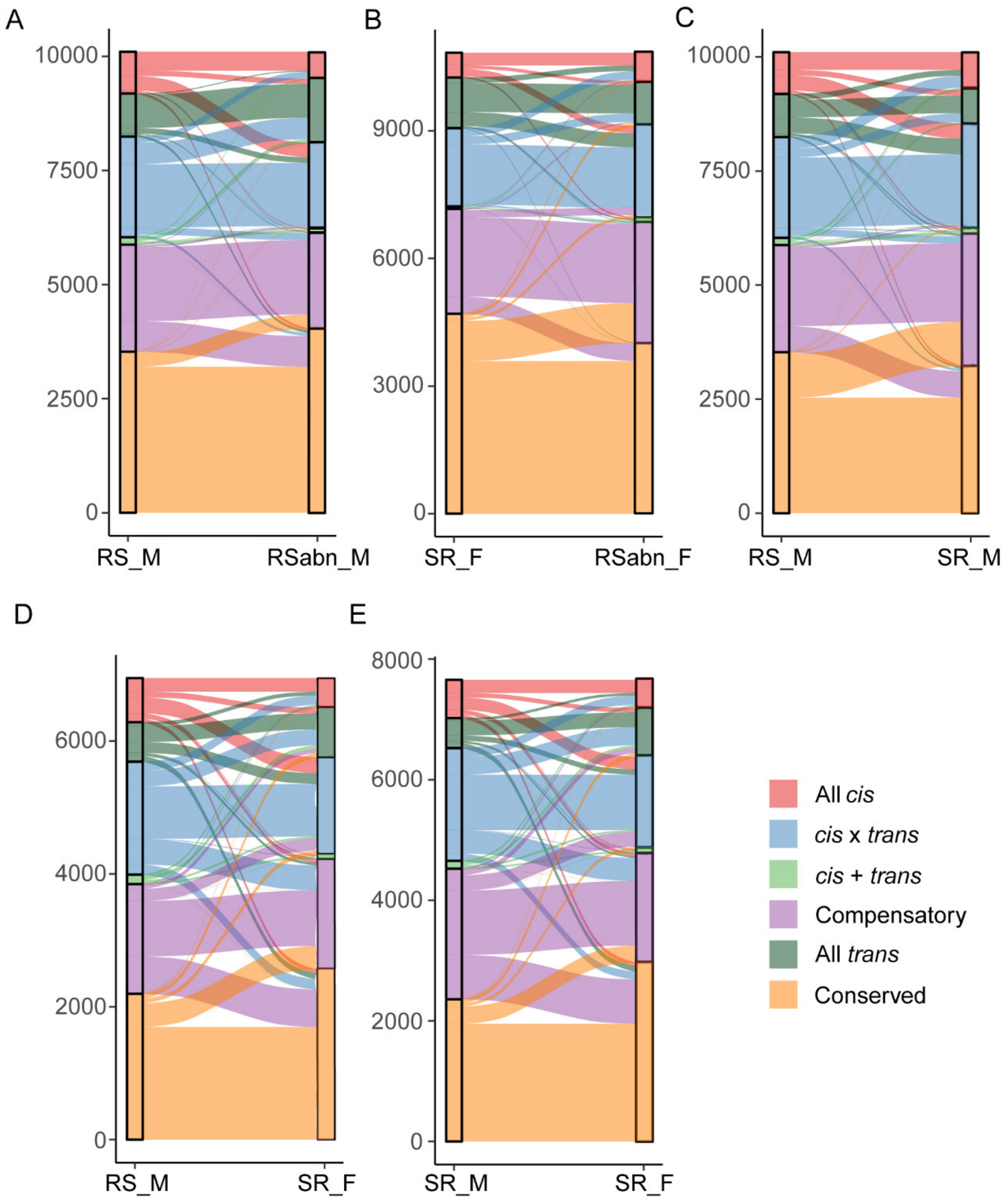

Figure S4. Alluvial plots showing changes in the numbers of genes inferred for each regulatory pattern through different F_1 hybrids.

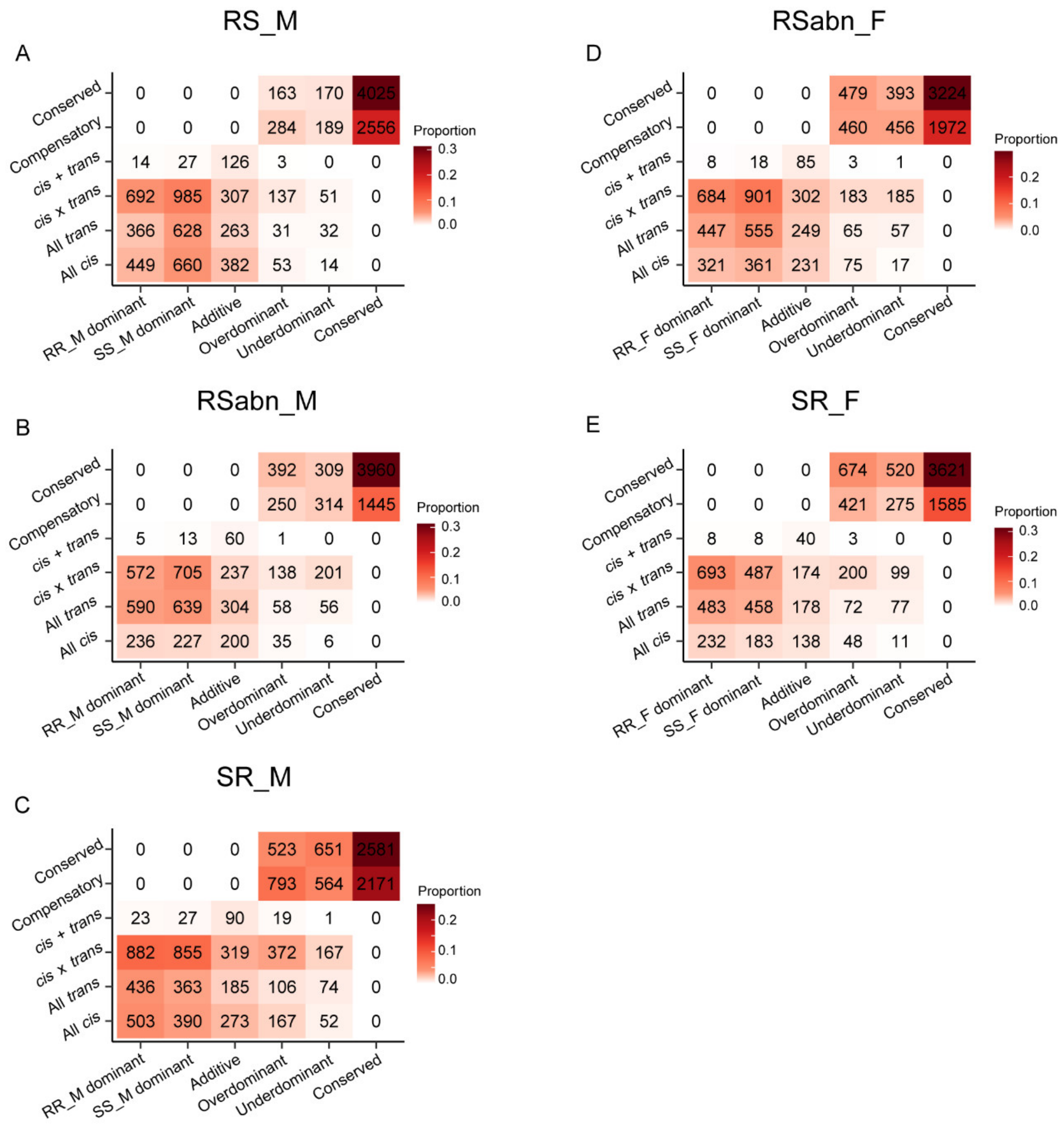

Figure S5. Relationships between regulatory patterns and inheritance modes. Heatmaps tally the number of genes with the same pair of classification in five groups of F_1 hybrids (RS_M (A), RSabn_M (B), SR_M (C), RSabn_F (D) and SR_F (E)), with inheritance modes on the *X*-axis and regulatory patterns on the *Y*-axis.
